# The relationship between nightmares and depression in adolescents: the effect of age and anxiety

**DOI:** 10.3389/fpsyt.2024.1408037

**Published:** 2025-01-20

**Authors:** Yuhang Li, Xiaorong Duan, Le Han, Ning Liu, Xueyang Han, Mingzhu Su, Tao Yang, Sha He, Rui Liu, Xinyu Gao, Yutong Xie, Jie Jiang, Laiqi Yang, Bin Xie, Xue Zou

**Affiliations:** ^1^ Department of Adolescent Mental Health, Xi’an International Medical Center, Xi’an, China; ^2^ Bio-X Research Institute, Shanghai Jiao Tong University, Shanghai, China; ^3^ Department of Psychology, Xi’an University of Physical Education, Xi’an, China

**Keywords:** nightmares, depression, age, anxiety, niacin skin flushing response

## Abstract

**Introduction:**

Nightmares and depression are prevalent issues among adolescents. This study explores the relationship between nightmares and depression, focusing on the mediating role of anxiety and the moderating effect of age

**Methods:**

A total of 210 adolescents aged 13 to 24 were surveyed using the Nightmare Distress Questionnaire (NDQ-CV), Self-Rating Anxiety Scale (SAS), and Self-Rating Depression Scale (SDS). They also underwent niacin skin flushing response (NSFR) testing to explore physiological correlations. A moderated mediation model was applied to assess the relationships between nightmares, anxiety, and depression. Spearman correlation analysis was used to analyze the relationship between nightmares and NSFR

**Results:**

Nightmares did not directly lead to depression, but anxiety served as a full mediator in this relationship. Age had no significant moderating effect. Additionally, a significant negative correlation between nightmares and NSFR was observed

**Conclusion:**

Nightmares contribute to anxiety, which can lead to depression in adolescents, suggesting that clinicians can identify and intervene with nightmares in adolescents to minimize the onset of psychological disorders. The study also highlights a possible connection between nightmares and NSFR, suggesting further research is needed to understand the physiological mechanisms

## 1 Introduction

Throughout history, dreams have been documented in human societies, with nightmares being a prevalent manifestation of dreaming (1). Freud’s theory is that the trauma experienced by the individual is expressed in the form of nightmares through the subconscious, and that the experience of these nightmares can cause harm to the human spirit (2). Nightmares, a common occurrence in dreams, have long received significant attention from the Western psychological community, resulting in a multitude of study findings. Studies have shown a correlation between nightmares and anxiety (3, 4). Li et al. found that, in contrast to the norm, the nightmares of college students with anxiety issues mainly included the appearance of dead or imagined characters, aggressive behavior, self-denial, and negative emotional content (5). Zhu et al. found a significant correlation between psychological damage caused by nightmares, anxiety, and depression, with participants having higher psychological damage showing higher levels of anxiety (6).Research has indicated that nightmares in adolescents may lead to anxiety and depression (7); however, the chain relationship among nightmares, anxiety, and depression has been reported infrequently.

Adolescence is a transitional period from childhood to adulthood (8), during which individuals undergo rapid physical development and begin to form a stronger sense of self and personality. This stage is marked by numerous challenges, making teenagers more vulnerable to psychological, emotional, and behavioral difficulties (9). Unresolved psychological issues during adolescence are likely to impact future development. In China, at least 30 million of the 367 million children and adolescents under 18 years old suffer from various emotional and behavioral problems (10). According to a national mental health survey, the prevalence of depressive symptoms among adolescents rises from 7.4% in early adolescence to 25.0% in late adolescence (11). One study reported that adolescents aged 13 to 17 had significantly higher self-rating depression scale (SDS) scores and prevalence of depressive symptoms compared to those aged 18 to 22 (12). This highlights a significant difference in depression levels across different age groups. With the adolescents’ physical and psychological changes, it is crucial to investigate whether different ages can help them cope with negative emotions more effectively.

Anxiety is one of the most prevalent psychological issues in modern society. When faced with significant challenges or difficulties, individuals often experience anxiety, which, if not addressed promptly and effectively, can escalate into a more severe anxiety disorder (13). Similarly, depression is another common psychological condition. According to recent data from 2022, 95 million people in China suffer from depression, representing approximately 3-5% of the global population (14). Traditionally, depression has been measured using self-report scales, but in recent years, the niacin skin flushing response (NSFR) test has emerged as a valid physiological measure. NSFR is a reaction to niacin that activates the phospholipase A2 (PLA2) to release arachidonic acid (AA) from membrane phospholipids, which is then converted into prostaglandins, leading to the flushing response (15). Research has found that the NSFR in adolescents with depression is significantly blunted, demonstrating its potential as a diagnostic biomarker (16). Moreover, it is reported that NSFR is negatively correlated with depressive and anxious symptoms, supporting the role of NSFR in mental health assessment (17). In addition, niacin is converted to nicotinamide in the body, playing a critical role in various biochemical processes, particularly in the synthesis and metabolism of neurotransmitters like serotonin and dopamine, which regulate mood. Blunted NSFR is considered a marker of niacin deficiency or dysfunction of cell membrane AA metabolism (18), which may coincide with neurotransmitter imbalances, contributing to the onset of depressive symptoms. Establishing a link between nightmares and NSFR could help to understand the physiological basis of nightmares and provide new ideas for early intervention to prevent depression.

In summary, this study aims to investigate the chain relationship between nightmares, anxiety, and depression, as well as the moderating role of age in this relationship. Additionally, the correlation between nightmares and NSFR was preliminarily explored.

## 2 Materials and methods

### 2.1 Participants

A total of 210 adolescents (June-August 2023) visiting a hospital was selected for the study. The Monte Carlo power analysis simulation was used for estimating sample size. Based on the correlation between nightmares, anxiety, and depression in our previous small sample study (N = 30), the minimum sample size needed to achieve a power of 0.95 was 64. The confidence level was set as 95%.In order to make the data more accurate and combined with the actual situation of the hospital, we actually recruited 210 cases. According to the age segmentation established by the World Health Organization, young people are defined as aged 10-24 years, youth as 15-24 years, and adolescents as 10-19 years (19). The adolescents selected for this study were aged between 13 and 24 years with an average age of 17.98 ± 3.24 years. The study included 98 males and 112 females. The study complied with the principles of the Declaration of Helsinki and was approved by the Medical Ethics Committee of Xi’an International Medical Center Hospital(202354).All study participants consented to the use of the questionnaire data for scientific research. The inclusion criteria were: 1) adolescents hospitalized for psychological issues. The exclusion criteria included: 1) Serious physical and medical history; 2) no history of nightmares; 3) unwillingness to cooperate for personal reasons.

### 2.2 Measurement tools

Nightmare Distress Questionnaire - Chinese version (NDQ-CV): NDQ-CV (20) was used to evaluate the patient’s nightmare distress. The NDQ-CV consists of 14 items (12 original NDQ items, 1 modified NDQ item, and 1 new item). Each item is rated on a five-point scale, 1= never, 2 = few, 3 = sometimes, 4 = often, 5 = always. The NDQ-CV is a translated version of the Nightmare Distress Questionnaire (NDQ), which has been validated for reliability and validity among normal adolescents. In a large sample of common Chinese adolescents, the internal consistency reliability of the NDQ-CV was 0.9 (21).

Self-Rating Anxiety Scale (SAS): The (SAS) (22) designed by Zung in 1971 was used to assess anxiety levels. The SAS is a 20-item scale, with each item answered on a four-point scale to assess the subjective feelings of patients with anxiety. In this study, the Cronbach’s α coefficient for this scale was 0.846, indicating good reliability.

Self-Rating Depression Scale (SDS): The SDS (23)was developed by Zung in 1965 as a self-assessment tool to assess the levels of depression. The SDS is a self-administered survey with high reliability, validity, and simplicity. The Cronbach’s α in this study is 0.85, indicating good reliability. According to a review of related literature, there are over 20 different self-rating depression scales. Some scales are specifically designed for children, such as Kovacs’ Children’s Depression Inventory (CDI), while others are adaptations of adult self-rating scales, such as the revised Beck Depression Inventory (BDI). SDS has been widely used in psychiatric clinics both domestically and internationally (24). Factor analysis has shown that the SDS has good construct validity when used among adolescents. Therefore, the SDS is suitable for assessing depressive symptoms in Chinese adolescents.

NSFR diagnostic instrument: Data are obtained from the diagnostic instrument using the skin patch method (Shanghai Tianyin Biological Technology Ltd., Shanghai, China) (25). The instrument included six different concentrations of aqueous methyl nicotinate (60, 20, 6.67, 2.22, 0.74, and 0.25mM), administered through a six-hole liquid carrier patch. Firstly, the instrument is capable of automatically aspirating 0.05 mL of aqueous methyl nicotinate at six different concentrations and dispensing it into the six corresponding holes of a patch. The patch was then placed in contact with the inner forearm of the subjects for one minute. Subsequently, the subjects’ arms were positioned in an imaging device, which automatically captured images every ten seconds for a duration of ten minutes, with results reported by computer. Reference interval: ≥3035—normal, [1825.6,3035)—low risk, [930.7,1825.6)—intermediate risk, and <930.7—high risk.

### 2.3 Study procedure

Nurses distributed the NDQ-CV, SAS, and SDS questionnaires to all participants in person. All participants completed the scale and NSFR tests with the help of nurses.

### 2.4 Statistical analysis

**Figure 1:**
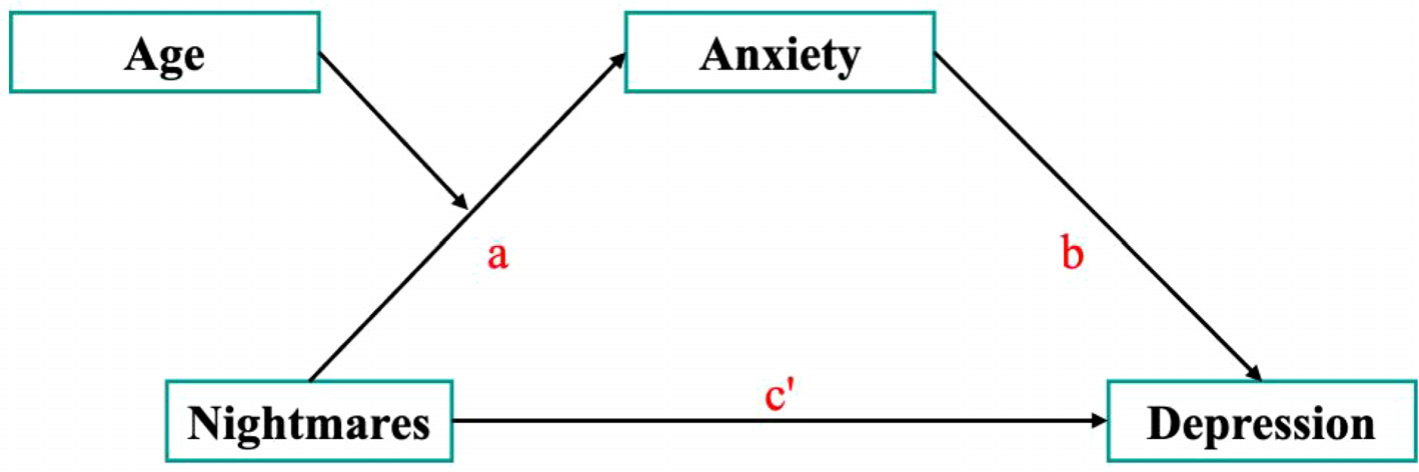
Schematic model of anxiety as the mediator between nightmares and depression.

All statistical analyses were performed using SPSS Statistics (version 26.0; IBM Corp).The correlations of study variables (nightmares, age, anxiety, and depression) were analyzed by Pearson correlation analyses. Firstly, we examined the regression analysis of the independent variable (nightmares) on the mediator variable (anxiety) (path a), while considering the moderating effect of age (**Figure 1**). Subsequently, we analyzed the regression of nightmares (path c’) and anxiety (path b) on depression. If the 95% confidence interval (CI) for the indirect effect (path a*b) did not include zero, this indicated a significant mediating effect. Building on this, if both path a and path b were significant while path c’ was not, this suggested a complete mediation effect. Conversely, if path c’ was significant, the possibility of partial mediation or suppression effects was considered based on the coefficients of path a and path b. Finally, the correlation between nightmares and NSFR was analyzed by Spearman correlation analysis.

## 3 Results

### 3.1 Correlation analysis of variables

To further examine the relationships between nightmares, age, anxiety, and depression, a correlation analysis was conducted. **Table 1** presents the means, standard deviations, and correlation coefficients between the variables. The results show significant positive correlations between nightmares and anxiety (r = .616, p <.01) as well as between nightmares and depression (r = .491, p <.01). Additionally, anxiety and depression exhibit a strong positive correlation (r = .831, p <.01). However, age shows no significant correlation with the other variables.

**Table 1:** Means, standard deviations, and correlation coefficients between variables.

Variables	M	SD	1	2	3	4
1. Nightmares	33.55	11.18	1			
2. Age	17.98	3.24	-.056	1		
3. Anxiety	58.00	12.42	.616^**^	.085	1	
4. Depression	66.26	14.21	.491^**^	.087	.831^**^	1

**p<.01, N = 210.

In addition, to explore whether adolescence and early adulthood exhibit different psychological patterns. Individuals aged 13 to 17 were categorized as minors, while those aged 18 to 24 were classified as adults. Independent sample t-tests were conducted to compare the nightmare and depression scale scores between these two groups. The results showed that the minor group had slightly lower mean scores for both nightmares and depression compared to the adult group (**Table 2**). However, the differences were not statistically significant (p >.05), indicating that there is no substantial variation in the psychological conditions between these age groups in terms of nightmares and depression.

**Table 2:** Differences in nightmare and depression scale scores among different age groups.

	Group	M ± SD	t	P
Nightmares	Minors (n=112)	32.96 ± 10.66	-.553	.581
	Adults (n=98)	33.94 ± 11.98		
Depression	Minors (n=112)	64.26 ± 15.68	-1.795	.074
	Adults (n=98)	68.31 ± 11.64		

### 3.2 Analysis of the mediation and moderating effects

As shown in **Table 3**, the model results indicated a significant positive effect of nightmares on anxiety (b = .697, p <.001), while the interaction term for nightmares and age (nightmares * age) did not have a significant impact on anxiety (b = –.036, p >.05), suggesting no moderating effect of age. Consequently, further simple slope analyses were unnecessary. Anxiety was found to have a significant positive effect on depression (b = .975, p <.001), whereas nightmares themselves did not significantly affect depression (b = –.043, p >.05) (**Table 4**). Thus, anxiety fully mediated the relationship between nightmares and depression, with a mediation effect value of 0.679 and a 95% CI of [0.557, 0.791], confirming the absence of zeros. Additionally, the findings showed that nightmares do not directly influence depression.

**Table 3:** Predictions of the mediating variable anxiety.

	Coeff	*t*	*p*	df	LLCI	ULCI
Nightmares	.697	11.635	.000	206	.579	.815
Nightmares* Age	-.036	-1.886	.061	206	-.0743	.002

* refers to the interaction term of nightmare by age.

**Table 4:** Predictions of the dependent variable depression.

	Coeff	*t*	*p*	df	LLCI	ULCI
Nightmares	-.043	-0.686	.494	206	-.165	.080
Anxiety	.975	17.396	.000	206	.864	1.085

### 3.3 Correlation analysis of nightmares with NSFR

After assigning participants to high, medium, and low groups according NSFR, the Spearman rank correlation analysis revealed a significant negative correlation between nightmares and NSFR (b = –.470, p <.01, **Table 5**).

**Table 5:** Correlation between nightmares and NSFR.

		Nightmares	NSFR
Nightmares	Correlation coefficient	1	-.470^**^
	Sig		.001

**p<.01, N = 210.

## 4 Discussion

### 4.1 Effect of age and anxiety

This study investigates the relationship between nightmares and depression, focusing on the roles of age and anxiety. The findings indicate that nightmares do not directly cause depression but that anxiety fully mediates the relationship. Nightmares typically involve terrifying or traumatic experiences that manifest in the subconscious during sleep (26). While these experiences are not real and do not cause direct harm, they can provoke significant anxiety. If this anxiety is prolonged and unresolved, it can eventually lead to depression, with sustained low mood being a key contributing factor (27).

The study also found that age did not have a moderating effect. This may be because the sample consisted of adolescents who were relatively close in age, limiting the ability to detect differences in coping mechanisms across age groups. The psychological immaturity of adolescents might hinder their ability to effectively manage the anxiety caused by nightmares. Individual differences in coping abilities, strategies, and personality traits could lead to varied responses to nightmares, influencing the development of anxiety and depressive symptoms.

In addition, the environment in which adolescents live and the pressures they face—such as academic stress, family dynamics, and social challenges—play crucial roles in shaping the relationship between nightmares, anxiety, and depression. These factors may be widespread among adolescents of all ages, which could explain the weak moderating effect of age. This study finds that nightmares’ psychological impact may persist with age unless properly addressed. It is important for parents and healthcare professionals to communicate and intervene in a timely manner. For instance, when adolescents experience anxiety due to nightmares, doctors could provide interventions like verbal reassurance or music-based (28) during their morning rounds, which could help reduce anxiety and prevent the development of depression.

The theoretical foundation for exploring age as a moderating factor in the relationship between nightmares, anxiety, and depression remains underdeveloped. Initially, it was hypothesized that older adolescents might be better equipped to manage the emotional distress caused by nightmares due to cognitive and emotional maturity. However, the lack of significant findings in this study suggests that the age range of the participants may have been too narrow to observe such effects. Adolescence is a developmental stage marked by rapid psychological, emotional, and physical changes, but individual differences in maturity can be subtle within a specific age bracket.

Future research could benefit from including a broader age range and examining how developmental milestones, such as the acquisition of emotional regulation skills and resilience, influence the relationship between nightmares and anxiety. This would allow for a more nuanced understanding of whether age moderates how adolescents process and cope with nightmares and whether age-related factors such as emotional maturity and stress-coping mechanisms can mitigate the risk of developing depression.

### 4.2 Relationship between nightmares and NSFR

The relationship between nightmares and NSFR has received limited support in conventional medical theories. Blunted NSFR is a biomarker for adolescent depression (29). The correlation between nightmares and NSFR revealed in this study provides further physiological evidence for the association of nightmares with depression, although the underlying mechanisms require further investigation.

It is noteworthy that blunted NSFR is thought to be associated with reduced levels of membrane AA, an abundant polyunsaturated fatty acids (PUFA) in the brain (30). Studies indicate that endocannabinoids derived from AA, such as anandamide and 2-arachidonoylglycerol, play a modulatory role in multiple neurobiological processes (31). It has been reported that the endocannabinoid system might regulate emotional states and dreams in humans and animal models (32). The significant association between nightmares and NSFR may imply that physiological changes associated with blunted NSFR have already occurred during the nightmare phase. In other words, adolescents with frequent nightmares may have abnormal AA metabolism, which causes disorders of the endocannabinoid system. Importantly, preliminary studies suggest that treatment with cannabinoids may reduce nightmares (33). In addition, randomized controlled trial has reported that supplementation of PUFA improves depressive symptoms and enhances NSFR in adolescents with depression (34). Therefore, for adolescents with blunted NSFR or frequent nightmares, PUFA-rich foods such as fish, eggs, and poultry are recommended to prevent the onset of depression.

### 4.3 Shortcomings of the study

The relationship between adolescent nightmares, anxiety, and depression is a complex psychological process influenced by various factors, including sleep duration and substance use. Adequate sleep is crucial for adolescents’ mental health, as insufficient sleep can disrupt nervous system function, increasing the risk of anxiety and depression (35). For adolescents who frequently experience nightmares, these disturbances can further reduce sleep quality, thereby exacerbating anxiety and depressive symptoms. Additionally, both excessively long and short sleep durations can negatively impact mental health.

Moreover, adolescence is a peak period for substance use, including drinking and smoking (36). These harmful habits are strongly correlated with depressive symptoms. Adolescents who experience frequent nightmares may resort to these substances to cope with stress and anxiety. However, while smoking and drinking may offer temporary relief, they often worsen depressive symptoms in the long term.

In summary, the relationship between adolescent nightmares, anxiety, and depression is shaped by various contributing factors, such as sleep patterns and substance use, which must be considered to fully understand their impact in future research.

Additionally, there are several limitations in this cross-sectional study: (1) They can only identify associations at a single point in time, without determining causality. (2) They cannot track changes in the frequency or severity of nightmares or changes in NSFR over time. (3) While statistical analyses can control for confounding factors, the absence of temporal data makes it challenging to fully eliminate all potential confounders.

For future longitudinal studies, the following recommendations are made: (1) To validate the causal relationships among nightmares, anxiety, and depression of adolescents by tracking these three variables at different time points. (2) Multiple time points could be established to track the correlation in nightmares and NSFR over time. (3) Track the developmental trajectories of adolescents’ psychological states following the recommendation of dietary supplementation with PUFAs. In addition, future studies should expand sample sizes and ensure a more representative population by including participants from different regions, age groups, and genders to improve the reliability and generalizability of findings. Since individual differences in nightmares and NSFR may vary, future studies should consider subgroup analyses to better understand these relationships.

## 5 Conclusion

Nightmares contribute to anxiety, which can lead to depression in adolescents, with no significant improvement observed with age. For adolescents with frequent nightmares, especially those accompanied by NSFR blunting, timely intervention may reduce depressive symptoms.

## Data Availability

The original contributions presented in the study are included in the article/supplementary material. Further inquiries can be directed to the corresponding authors.
